# Vaccine-induced thrombotic thrombocytopenia following coronavirus vaccine: A narrative review

**DOI:** 10.1016/j.amsu.2021.102988

**Published:** 2021-10-30

**Authors:** Syed Hassan Ahmed, Taha Gul Shaikh, Summaiyya Waseem, Nashwa Abdul Qadir, Zohaib Yousaf, Irfan Ullah

**Affiliations:** a Dow University of Health Sciences, Karachi, Pakistan; b Department of Internal Medicine, Hamad Medical Corporation, Doha, Qatar; c Kabir Medical College, Gandhara University, Peshawar, Pakistan

**Keywords:** COVID-19, COVID-19 vaccine, Vaccine induced thrombotic thrombocytopenia, Vaccine induced immune thrombotic thrombocytopenia, Thrombotic thrombocytopenia, VITT

## Abstract

The novel coronavirus pandemic has taken a toll on the global healthcare systems and economy. Safety precautions, along with vaccination, are the most effective preventive measures. The global vaccination program against COVID-19 has dramatically reduced the number of deaths and cases. However, the incidence of thrombotic events and thrombocytopenia post-COVID-19 vaccination known as vaccine-induced thrombotic thrombocytopenia has raised safety concerns. This has led to an element of vaccine hesitancy. The exact mechanism for vaccine-induced thrombotic thrombocytopenia is unknown. Although the incidence of thrombosis associated with COVID-19 vaccination is low, it still requires attention, especially in older people, smokers, and people with preexisting comorbidities. This study aims to review the pathophysiology, diagnosis, and management of vaccine-induced thrombotic thrombocytopenia, to provide a concise and comprehensive update.

## Highlights

• The global vaccination program against COVID-19 has greatly reduced the number of deaths and cases.
• This review highlights the potential pathophysiology, clinical diagnosis, and management of COVID vaccine associated VITT.
• The escalating incidence of such cases has raised concerns regarding the safety profile of available vaccines.
• Despite several cases of VITT being reported, the benefits of vaccines continue to markedly outweigh the rare ramifications.

## 1 Introduction

The Severe Acute Respiratory Syndrome Coronavirus (SARS-CoV-2) cases were initially reported in Wuhan, China, towards the end of 2019. Following its extensive spread, the World Health Organization (WHO) declared COVID-19 a pandemic in March 2020 [1]. To the date, April 16, approximately 207 million confirmed cases have been reported, and 4.3 million deaths [2].

Coordinated global efforts led to the development of COVID-19 vaccines, followed by emergency use authorization within nine months of the pandemic [3]. These vaccines are now widely available for public administration [4]. The vaccines are safe and effective in preventing severe infection, hospitalization, and death [5,6]. To date, 4.4 billion vaccine doses have been administered [2]. The common adverse effects following COVID-19 vaccination are injection site pain and transient, self-limited systemic symptoms like headache, fever, myalgias, etc. [7].

Recently, a more severe adverse effect, thrombocytopenia with or without thrombosis, has been reported following SARS-CoV-2 vaccination. Thrombocytopenia is a medical condition characterized by platelets lower than 150,000/microliter and is associated with a risk of bleeding and thrombosis [8]. Such reports have raised concerns over the safety profile and hesitancy towards the available vaccines [9]. The term “Vaccine-Induced Thrombotic Thrombocytopenia” describes post-vaccination thrombocytopenia cases. VITT is characterized by thrombosis at unusual sites and thrombocytopenia following vaccination [9].

While VITT has been associated with both mRNA and viral vector vaccines, its prevalence is higher in viral vectored vaccines [7]. Following the incidence of 30 thromboembolism cases in March 2021, Oxford/AstraZeneca (AZD1222) was transiently suspended in numerous European countries [10]. Later the pharmacovigilance risk assessment committee (PRAC) of the European medical agency (EMA) reviewed all cases and declared thrombosis and thrombocytopenia as rare adverse effects of AZD1222. However, based on risk-benefit assessment, the vaccine was later declared safe for use [11]. Owing to a similar reason, in April 2021, Johnson & Johnson's Janssen (Ad26.CoV2·S) administration was also temporarily suspended [12].

Herein, we review the association between SARS-CoV-2 vaccines and VITT. This review evaluates the potential pathophysiology and clinical approach to diagnoses and management of VITT.

### 1.1 Literature review

**Fig. 1:**
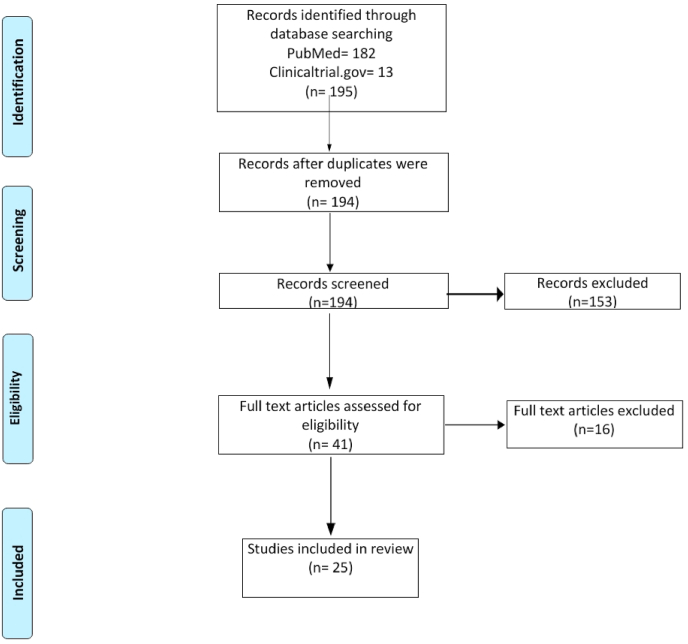
Prisma flowchart.

The work has been reported in line with the PRISMA 2020 criteria [13]. Two authors (SHA, SW) dependently conducted a thorough literature search over PubMed and Clinicaltrials.gov from inception till August 16, 2021, without any language restriction. To achieve comprehensive results, search string comprised of keywords, “SARS-CoV-2 Vaccine”, “Coronavirus Vaccine,” “Corona Vaccine,” “COVID-19 Vaccine”, “thrombotic thrombocytopenic,” “Vaccine-Induced Thrombotic Thrombocytopenia,” “VITT,” “thrombocytopenia,” “reduced platelet count,” using BOOLEAN operators. Synonyms, related terms, and spelling variants were also engaged. All relevant case reports, case series, cohort studies, editorials, and correspondences were reviewed. Any discrepancies were resolved via discussion with a third reviewer (IU). The results of the literature search are shown in Fig. 1. Following studies selection, two independent authors (TGS, NAQ) extracted all the relevant data into a table comprising of author's name, patient's age, and sex, past medical history, presenting complaint, laboratory findings, radiological findings, treatment interventions, and outcome. Any discrepancies were resolved by discussion with a third reviewer (IU). All significant findings are summarized in Table 1.

**Table 1:** A tabulation of the outcomes of literature review of VITT following SARS-CoV-2 vaccination.

Author	Sex and Age	Past Medical history	Presenting Complaint	Vaccine administered	Laboratory findings	Radiological findings	Intervention	Outcome
Al Maqbali et al. [55]	59 y/o Female	Type 2 diabetes mellitus, osteoarthritis, and COVID-19 pneumonia in September 2020, OCP	Sudden onset left leg pain 7 days after receiving her first dose.	Pfizer-BioNTech mRNA	Platelet = 182 × 10^9^/L D-dimer = 24 mg/L	Bifurcation of the pulmonary trunk and main pulmonary arteries emboli extending to the lobar segmental and subsegmental branches	Rivaroxaban 2 × 15 mg daily for 21 days, followed by rivaroxaban 20 mg daily for a total of 3 months	Recovered
Muir et al. [56]	48 y/o Male	N/A	3 days history of malaise and abdominal pain	Ad26.COV2. S vaccine (Johnson & Johnson/Janssen)	Platelet = 13,000/mm^3^ D-dimer = 117.5 mg/Liter	Cerebral venous sinus thrombosis involving the right transverse and straight sinuses and extensive splanchnic vein thrombosis	Argatroban & IVIG at a dose of 1 g/kg of ideal body weight	Critically ill at the time of the report
Sheikh et al. [57]	50 y/o Male	N/A	Headache, vertigo, and vision changes	ChAdOx1 nCoV-19 (AstraZeneca)	N/A	Central venous sinus thrombosis (CVST) in transverse and sigmoid sinuses	Desirudin, IVIG at 1 g/kg/hour and Prednisolone at 1 mg/kg daily	Recovered
Ramdeny et al. [58]	54 y/o Male	Rare congenital limb malformation	7-day history of worsening headache, bruising and unilateral right calf swelling	ChAdOx1 nCoV-19 (AstraZeneca)	Platelet = 34. x 10^9^/L D-dimer = 6000 ng/mL	Extensive cerebral venous sinus thrombosis	Therapeutic IVIG and anticoagulation	Recovered
Bano et al. [49]	53 y/o Female	N/A	Worsening headache and weakness of the right arm and leg	ChAdOx1 nCoV-19 (AstraZeneca)	Platelet = 24 × 10⁹/L D-dimer = 5620 ng/mL	Cerebral Venous sinus thrombosis	Three units of platelets were transfused before urgent neurosurgical intervention	Death
Bano et al. [49]	61 y/o Female	N/A	3-day history of progressive dyspnea, pain, and swelling in the right leg	ChAdOx1 nCoV-19 (AstraZeneca)	Platelet = 25 × 10⁹/L D-dimer = 9376 ng/mL	Bilateral PE with right heart strain	One unit of platelets, LMWH, was given twice. After which, anticoagulation was switched to treatment dose fondaparinux. Further platelet transfusion was withheld. The patient was treated with IVIG 1 g/kg single dose and pulsed dexamethasone 20 mg once daily for 4 days	Recovered
Wiedmann et al. [59]	42 y/o Female	N/A	Severe headaches, nausea, vomiting, fluctuating level of consciousness, and right-sided hemiparesis	ChAdOx1 nCoV-19 (AstraZeneca)	N/A	Left transverse sinus and sigmoid sinus cerebral sinus vein thrombosis (CSVT) and cortical vein thrombosis	IV methylprednisolone (1 mg/kg) daily and IVIG (1 g/kg) for 2 days	Death
Wiedmann et al. [59]	37 y/o Female	N/A	2-day history of headaches, fever, transient numbness in the right foot, and right-sided visual disturbance	ChAdOx1 nCoV-19 (AstraZeneca)	N/A	CSVT in the left transverse and sigmoid sinus and left occipital CSVT	Urgent suboccipital craniectomy was performed and cerebellar herniation encountered during surgery	Death
Wiedmann et al. [59]	39 y/o Female	N/A	Abdominal pain and headaches	ChAdOx1 nCoV-19 (AstraZeneca)	Platelet = 119 × 10^9^/L	Small cerebellar hemorrhage. CSVT in the inferior sagittal sinus, vein of Galen and straight, right transverse and sigmoid sinuses. Bilateral segmental pulmonary emboli, thrombosis in uterine veins.	IVIG, steroids, warfarin	Recovered
Wiedmann et al. [59]	54 y/o female	N/A	Numbness of left-sided limbs 6 days post-vaccination, left-sided paralysis and facial nerve palsy.	ChAdOx1 nCoV-19 (AstraZeneca)	N/A	CSVT in nearly all major venous sinuses	Methylprednisolone (1 mg/kg) and IVIG (1 g/kg) for 2 days and decompressive hemicraniectomy	Death
Ruhe et al. [51]	84 y/o Female	N/A	Partial hemiplegia, scattered petechiae, and severe arterial hypertension.	Pfizer-BioNTech mRNA	Platelet count = 45 × 10^9^/L	Multiple subacute emboli without vessel occlusion.	Corticosteroid and plasma exchange therapy (PEX) with fresh frozen plasma. Rituximab at day 2 as second corticosteroid	Recovering
Gesseler et al. [60]	47 y/o Female	N/A	Progressive headache 7 days after the first dose	ChAdOx1 nCoV-19 (AstraZeneca)	Platelet = 9 × 10^9^/L D-dimer >35.2 mg/L	Large-scale sinus thrombosis	IVIg at 1 g/kg, Argatroban and corticosteroids. Platelet therapy was administered before the decompressive surgery. During operation, artificial hemostyptics and transfusions were done.	Death
Gesseler et al. [60]	50 y/o Female	N/A	Progressive headache 10 days after first dose	ChAdOx1 nCoV-19 (AstraZeneca)	Platelet = 24 × 10^9^/L D-dimer >35.2 mg/L	Large-scale sinus thrombosis	IVIg at 1 g/kg, Argatroban and corticosteroids. Platelet therapy was administered before the decompressive surgery. During operation, artificial hemostyptics and transfusions were done.	Death
Gesseler et al. [60]	44 y/o Female	N/A	Progressive headache 12 days after the first dose	Ad26.COV2. S vaccine (Johnson & Johnson/Janssen)	Platelet = 48 × 10^9^/L D-dimer >35.2 mg/L	Large-scale sinus thrombosis	IVIg at 1 g/kg, Argatroban and corticosteroids. Platelet therapy was administered before the decompressive surgery. During operation, artificial hemostyptics and transfusions were done.	Death
Patel et al. [15]	33 y/o Male	N/A	Back pain, hematuria, headache, and right lower leg pain for 1 week	ChAdOx1 nCoV-19 (AstraZeneca)	D-dimer >20 mg/L Anti-PF4 antibodies were positive	PE in left pulmonary artery	IV Argatroban, IVIG, and warfarin	Recovered
Patel et al. [15]	28 y/o Male	N/A	Back pain and lower limb weakness	ChAdOx1 nCoV-19 (AstraZeneca)	Elevated D-dimers and positive anti-PF4 antibodies	Bilateral PEs and left proximal DVT	IV Argatroban, IVIG, and warfarin	Recovered
Patel et al. [15]	61 y/o Male	N/A	Exertional dyspnea and pleuritic chest pain	ChAdOx1 nCoV-19 (AstraZeneca)	Elevated D-dimers and positive anti-PF4 antibodies	Bilateral PEs	IV Argatroban, IVIG, and warfarin	Recovered
Suresh et al. [17]	27 y/o Male	N/A	Intermittent headaches associated with eye floaters	ChAdOx1 nCoV-19 (AstraZeneca)	Platelets = 12 × 10^9^/L *Anti*-P4 antibodies were positive	Cerebral venous sinus thrombosis.	IVIg (1 g/kg) once a day, Dabigatran, Idarucizumab, Prednisolone once daily (1 mg/kg) with proton pump inhibitors cover	Death
Mehta et al. [16]	32 y/o Male	N/A	Thunderclap headache, subsequent left-sided incoordination, and hemiparesis	ChAdOx1 vaccine (AZD1222, Vaxzevria)	Platelets = 30 × 10^9^/L	Superior sagittal sinus and cortical vein thrombosis	No treatment since the condition continued to deteriorate	Death
Mehta et al. [16]	25 y/o Male	Primary sclerosing cholangitis and migraines	Photophobia, neck stiffness, visual disturbances, petechial rashes, and gum bleeding	ChAdOx1 vaccine (AZD1222, Vaxzevria)	Platelets = 19 × 10^9^/L Positive *anti*-P4 antibodies	Superior sagittal sinus thrombosis with cortical veins involvement.	Intravenous unfractionated heparin, platelet transfusions, IV dexamethasone, IVIG, and intravenous levetiracetam	Death
Xie et al. [50]	23 y/o	N/A	Chest pain and breathlessness	N/A	Platelets = 73 × 10^9^/L D-dimer = 17548 μg/L	Pulmonary emboli, right ventricle thrombus, and splenic vein thrombus	Apixaban, intubation, ventilation, plasma exchange, IV methylprednisolone, and heparin infusion	Recovered
Sørensen et al. [22]	33 y/o Female	Migraine	Headache and general malaise	ChAdOx1 nCoV-19 (AstraZeneca)	Platelets = 51 × 10^9^/L Anti-PF4 antibodies were positive	Cerebral venous sinus thrombosis and portal vein thrombosis	Tinzaparin, Fondaparinux	Recovered
Dias et al. [61]	47 y/o Female	Iron deficiency anemia due to adenomyosis	Headache, nausea, and photophobia	Pfizer-BioNTech mRNA	Platelets = 34000/mL Anti-PF4 antibodies were negative	Thrombosis of superior sagittal, right lateral, transverse, sigmoid sinuses and jugular vein and left sigmoid sinus	Acetazolamide, enoxaparin 60 mg, and warfarin	Recovered
Dias et al. [61]	67 y/o Female	Multiple cerebral cavernous malformations, hypertension, diabetes, dyslipidemia, viral myocarditis, and depression	Right lower limb clonic movements, motor deficit, loss of consciousness, and headache	Pfizer-BioNTech mRNA	Platelets = 164000/mL Anti-PF4 antibodies were negative	Thrombosis of high convexity cortical veins, superior sagittal, right transverse, and sigmoid sinus and jugular vein	Levetiracetam 500 mg, enoxaparin 80 mg, dabigatran 150 mg	Recovered
Tiede et al. [18]	63 y/o Female	N/A	Headache, somnolence, dysphasia, right-sided hemiparesis, and arterial hypertension	ChAdOx1 vaccine (AZD1222, Vaxzevria)	Platelets = 27/nl D-dimer >35.2 mg/L Anti-PF4 antibodies were positive	Left transverse and sigmoid sinus thrombosis, cerebral venous sinus thrombosis	Heparin and eculizumab	Recovering
Tiede et al. [18]	67 y/o Female	N/A	Headache	ChAdOx1 vaccine (AZD1222, Vaxzevria)	Platelets = 40/nl D-dimer >35.2 mg/L Anti-PF4 antibodies were positive	Aortic arch thrombi and cerebral arterial embolism	Argatroban and IVIG	Recovered
Tiede et al. [18]	61 y/o Female	N/A	Fatigue	ChAdOx1 vaccine (AZD1222, Vaxzevria)	Platelets = 12/nl D-dimer >35.2 mg/L Anti-PF4 antibodies were positive	Splanchnic vein thrombosis	Argatroban, IVIG, alteplase, eculizumab	Recovering
Tiede et al. [18]	61 y/o Female	N/A	Headache, dysarthria, left-sided hemiplegia, conjugated gaze palsy	ChAdOx1 vaccine (AZD1222, Vaxzevria)	Platelets = 62/nl D-dimer> 35.2 mg/L Anti-PF4 antibodies were positive	Right internal carotid and middle cerebral artery (M1) thrombosis and cerebral arterial thrombosis	Argatroban and IVIG	Recovering
Guetl et al. [62]	50 y/o Female	N/A	Severe headache and severe back pain	ChAdOx1 nCoV-19 (AstraZeneca)	Platelets = 27 × 10^9^/L D-dimer >33 mg/L Anti-PF4 antibodies were negative	Multifocal thrombus in the pelvic region and embolus in the posterior–basal right lower lobe	IVIG, dexamethasone 40 mg, argatroban, and dabigatran	Recovered
Schultz et al. [46]	37 y/o Female	Pollen allergy	Headaches, fever, and visual disturbance	ChAdOx1 nCoV-19 (AstraZeneca)	Platelets = 22000 mm^3^ D-dimer> 35 mg/L	Thrombosis in the left transverse, left sigmoid sinuses, and cortical veins	Dalteparin, platelets, and decompressive craniotomy	Death
Schultz et al. [46]	42 y/o Female	Pollen allergy	Headache and drowsiness	ChAdOx1 nCoV-19 (AstraZeneca)	Platelets = 14000 mm^3^	Thrombosis in the left transverse left sigmoid sinuses and cortical veins	Dalteparin, platelet transfusion, IVIg 1 g/kg, methylprednisolone 1 mg/kg, and hemicraniectomy	Death
Schultz et al. [46]	32 y/o Male	Asthma	Back pain	ChAdOx1 nCoV-19 (AstraZeneca)	Platelets = 10,000 mm^3^	Thrombosis of portal vein branches	IVIg 1 g/kg, prednisolone 1 mg/kg, dalteparin	Recovered
Schultz et al. [46]	39 y/o Female	N/A	Headache and abdominal pain	ChAdOx1 nCoV-19 (AstraZeneca)	Platelets = 70000 mm^3^	Thrombosis of Inferior sagittal sinus, straight sinus, the vein of Galen, right transverse sinus, and right sigmoid sinus	IVIg 1 g/kg, prednisolone 1 mg/kg, dalteparin, warfarin	Recovered
Schultz et al. [46]	54 y/o Female	Hypertension	Headache and hemiparesis	ChAdOx1 nCoV-19 (AstraZeneca)	Platelets = 19000 mm^3^	Thrombosis of the cortical vein, superior sagittal vein, both transverse sinus and left sigmoid sinus	IVIg 1 g/kg, methylprednisolone 1 mg/kg	Death
Malik et al. [63]	43 y/o Female	Hyperlipidemia, anxiety, depression, obesity, obstructive sleep apnea, and gastroesophageal disease	Headache, fever, body aches, chills, mild dyspnea, and light-headedness	Ad26.COV2. S vaccine (Johnson & Johnson/Janssen)	Platelets = 27 × 10^9^/L D-dimer = 35.2 mg/L Anti-PF4 antibodies were positive	Pulmonary Embolism and Intracerebral thrombus	IVIg, fondaparinux, fioricet and topiramate	Recovered
Garnier et al. [14]	26 y/o Female	N/A	Nausea and headache	ChAdOx1 nCoV-19 (AstraZeneca)	Anti-PF4 antibodies were positive	Occlusion in middle cerebral artery	Corticosteroids, anticoagulants, and plasma exchange	N/A
Abadi et al. [20]	30 y/o Female	N/A	Headache, neck pain, lower extremity pain, and weakness	Ad26.COV2. S vaccine (Johnson & Johnson/Janssen)	Platelets = 80 × 10^3^/μL Anti-PF4 antibodies were positive	Acute deep vein thrombosis involving posterior tibialis and popliteal veins, obstructive thrombosis in right transverse sinus extending to right sigmoid sinus and jugular bulb, pulmonary embolism	Argatroban and Bivalirudin	Recovered
Agostino et al. [64]	54 y/o Female	N/A	Acute cerebrovascular accident	ChAdOx1 nCoV-19 (AstraZeneca)	Normal D-dimer	Deep vein thrombosis, acute basilar thrombosis	N/A	Death
Mauriello et al. [65]	48 y/o Female	Penicillin allergy, episode of thrombocytopenia in 2016. Postmortem analysis indicated pre-existing thrombocytopenia	Progressive headache, back pain, moderate right lower limb pain, and disseminated ecchymosis that required hospitalization on day 18	ChAdOx1 nCOV-19 AstraZeneca	Platelets = 32000/μL D-dimer = 10 mg/mL	Thrombo-embolic filling defects affecting the pulmonary artery, sigmoid transverse sinus thrombosis, right internal jugular vein thrombosis, right temporo-occipital intraparenchymal hemorrhage	Initially low molecular weight heparin, anti-hypertensive, oral double (dabigatran 110 mg/die + rivaroxaban 30 mg/die) anticoagulants, IV methylprednisolone, dabigatran antagonist, and a decompressive craniectomy	Death
Wolf et al. [21]	22 y/o Female	N/A	Shivering, fever, and headaches for two days, with spontaneous resolution Day 4: New frontally accentuated headaches Day 7: A self-limited generalized epileptic seizure occurred.	ChAdOx1 nCoV-19 (AstraZeneca)	Platelets = 75000/Ul D-dimer = 2590 ng/mL Anti-PF4 antibodies were positive	Superior sagittal sinus, left side transverse sinus, sigmoid sinus, and ascending cerebral veins thrombosis.	Endovascular rheolysis, 2 × 1000 mg levetiracetam (PO) daily for three months, 2 × 80 mg enoxaparin sodium (SC) daily for ten days, followed by direct oral anticoagulation with 2 × 150 mg dabigatran PO daily for six months.	Recovered
Wolf et al. [21]	46 y/o Female	N/A	Severe headaches eight days after the first dose	ChAdOx1 nCoV-19 (AstraZeneca)	Platelets = 60,000/ul Anti-PF4 antibodies were positive	Superior sagittal sinus, left-hand transverse sinus, sigmoid sinus thrombosis.	Endovascular rheolysis in two separate sessions, 2 × 80 mg SC Enoxaparin for 2 days then changed to 3 × 750 mg Danaparoid	Recovered
Wolf et al. [21]	36 y/o Female	N/A	Severe headaches seven days after the first dose, three days of fever and headache, acute somnolence, and right-hand hemiparesis	ChAdOx1 nCoV-19 (AstraZeneca)	Platelets = 92000/ul Anti-PF4 antibodies were positive	Straight sinus thrombosis, non-occlusive thrombus in the superior sagittal sinus	2250 IU danaparoid SC, endovascular rheolysis, 2 × 60 mg enoxaparin sodium SC daily for one week, followed by direct oral anticoagulation with 2 × 150 mg dabigatran PO daily for six months	Recovered
Bjørnstad-Tuveng et al. [19]	Female in her 30s	An uncomplicated birth 11 months prior with 1500 mL bleeding, mild preeclampsia treated with labetalol 100 mg. For the past 3 months, she used Duroferon 2 × 100 mg for iron deficiency and desloratadine 5 mg for allergies.	Headache after 7 days of vaccination. This was followed by a worsening headache, slurred speech, and uncoordinated walking and movement.	ChAdOx1 nCoV-19 (AstraZeneca)	Platelets = 37 × 10^9^/L D-dimer > 7.0 mg/L Anti-PF4 antibodies were positive	Postmortem examination revealed fresh small thrombi in the transverse sinus, frontal lobe, and pulmonary artery.	1 g of tranexamic acid intravenously, midazolam for seizure	Death
Tarawneh et al. [48]	22 y/o male	N/A	Petechia and gums bleeding 3 days post-vaccination	Pfizer-BioNTech mRNA	Platelets = 2 × 10^9^	N/A	Dexamethasone 40 mg daily for 4 days, platelet transfusion, and IVIG at 1 g/kg for 2 days	Recovered

N/A: Not Available, OCP: Oral contraceptives, IVIG: Intravenous Immunoglobulins, ITP: Immune thrombocytopenia, PE: Pulmonary embolism, CVST: Cerebral venous sinus thrombosis, IV: Intravenous, SC: Subcutaneous, PO: Per os LMWH: Low molecular weight heparin, DVT: Deep vein thrombosis.

### 1.2 Demographics

**Fig. 2:**
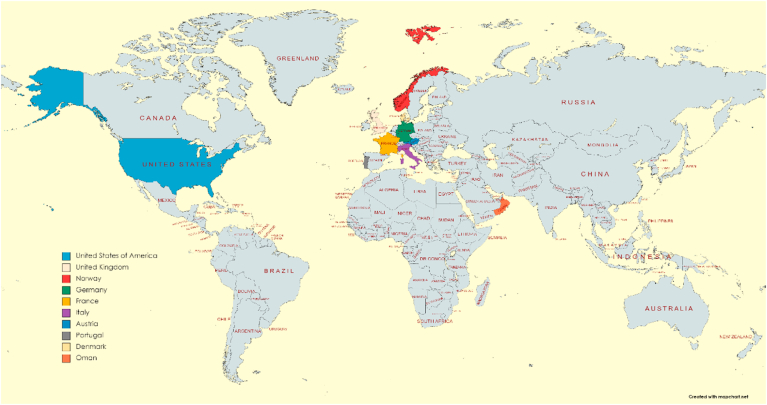
Geographical distribution of the reported cases.

The retrieved studies comprise data of 44 patients (32 females, 11 males, 1 not defined) with a mean age of 44.9 ± 14.3 years. The following figure (Fig. 2) depicts the geographical distribution of the reported cases around the globe, with the majority of cases arising in Europe. Based on these and future reporting, we can predict the potential spatial spread, geographical locations that may be more susceptible than others and this may help us establish links between different genetic and environmental factors, predisposing an individual to such consequences of vaccines.

### 1.3 Pathophysiology

The exact pathophysiology behind VITT is unclear. As shown in Table 1, most of the cases presented with thrombocytopenia, elevated D-dimer, and positive titers of IgG antibodies against platelet factor 4 (PF-4) [[14], [15], [16], [17], [18], [19], [20], [21], [22]]. Based on these findings, this syndrome is closely related to heparin-induced thrombocytopenia (HIT), a medical condition characterized by thrombocytopenia, and the presence of antibodies against the Heparin-PF4 complex [23].

HIT, an autoimmune reaction to heparin, involves the generation of IgG antibodies against the Heparin-PF4 complex. The Fc portion of these antibodies adheres to the complex, binds to the FcYRIIa receptors [24], and initiates platelets activation via intracellular signaling involving spleen tyrosine kinase [25]. This results in the release of microparticles and a procoagulant state [26,27]. Furthermore, clearance of activated and antibody-bound platelets by the reticuloendothelial system culminates in thrombocytopenia [28]. A prerequisite in the diagnosis of HIT includes a known recent exposure to heparin. A condition labeled “Autoimmune Heparin-Induced Thrombocytopenia (aHIT)” manifests with clinical and laboratory findings without any prior use of heparin [29]. Based on this resemblance, a comparison has been drawn between VITT and variants of aHIT [30], and hence, we may assume that a similar mechanism follows post-vaccination. However, the mechanism behind the generation of these antibodies is yet to be elucidated.

In HIT, the electrostatic interaction between positively charged PF4 and negatively charged heparin culminates in the formation of the Heparin-PF4 complex [31]. This phenomenon has also been observed with other negatively charged molecules like numerous polyphosphates [32], Polyvinyl phosphonate [33], nucleic acids [34], etc. According to Visentin et al. [33], numerous negatively charged molecules, spaced about 0.5 nm apart along the molecular backbone and of sufficient length, can form complexes with PF4 while being detectable by the antibodies. Hence, components of vaccines can be expected to play a crucial role in the generation of PF4-polyanions complex and antibodies against them. Moreover, environmental factors and genetic predisposition can exacerbate clinical presentation. For example, specific genotypes encoding FcRIIA have been associated with an increased risk of thrombosis in individuals with anti-PF4-polyanion antibodies [24].

Another postulated mechanism involves cross-reactivity of *anti*-SARS-CoV-2 spike protein antibodies that generates following SARS-CoV-2 vaccination with PF4. This may be attributable to molecular mimicry, a phenomenon whereby a certain degree of resemblance exists between the pathogens and the host's antigens [35]. Kanduc et al. [36] report massive homogeneity between the SARS-CoV-2 spike glycoprotein and human proteins, thus further strengthening this hypothesis. This structural resemblance can also explain the findings of thrombocytopenia [37] and anti-PF4 antibodies [38] in certain SARS-CoV-2 patients. However, the currently available literature suggests no evidence of cross-reactivity [38,39].

Zhang et al. [40] investigated the findings of thrombosis and thrombocytopenia in SARS-CoV-2 patients. They reported spike protein's ability to stimulate platelet activation and thrombus formation via the Mitogen-activated protein kinase (MAPK) pathway. Based on the findings [40], the generation of spike protein following vaccination can also play a pivotal role in inducing thrombocytopenia and thrombosis via spike protein-ACE2 interaction-induced platelets activation. However, it remains unanswered if similar interactions can be observed post-vaccination with vector or mRNA vaccines. Moreover, some evidence [41] reveals potential interactions between adenovirus particles and circulating platelets leading to platelet activation and aggregation. The possibility of such interactions in the case of viral vector-based vaccines cannot be ruled out and requires further investigation. Furthermore, as shown in Table 1, the findings of negative anti-PF4 antibodies in selective cases indicate involvement of a non-HIT like mechanism hence strengthening the above suggested hypothesis.

Future research should focus on potential interactions between spike proteins and platelets and the phenomenon of cross-reactivity. Another intriguing aspect of the higher prevalence of VITT among individuals vaccinated with viral vector-based vaccines needs to be investigated in the search for potential links. Development of thrombosis in selective individuals and incidence of rare site thrombosis like cerebral venous sinuses deserve equal attention for the exact pathophysiology to be elucidated. Lastly, the development of anti-PF4 antibodies only in certain VITT patients can also provide important clues in determining the pathogenesis.

### 1.4 Diagnosis

Following the escalation in reported thrombocytopenia and thrombosis cases post COVID-19 vaccination, the American Society of Hematology (ASH) reviewed all the reported cases and laid specific ground rules to diagnose this novel presentation. As per the ASH [42], cases meeting the following criteria can be identified as VITT:

a) Symptom onset 4–42 days post SARS-CoV-2 vaccination
b) Any venous or arterial thrombosis (often cerebral or abdominal)
c) Thrombocytopenia
d) Antibodies to platelet factor 4 (PF4) identified by enzyme-linked immunosorbent test (ELISA)
e) Markedly elevated D-dimer (>4 times upper limit of normal)

Individuals presenting with the complaints of severe headache, visual changes, abdominal pain, nausea, vomiting, back pain, shortness of breath, leg pain or swelling, petechiae, easy bruising, or bleeding, 4–42 days post-vaccination, must be evaluated critically for the condition mentioned above. Laboratory investigations, including CBC with platelet count, PF4 ELISA, d-dimer, fibrinogen, and imaging techniques for thrombosis, can play a crucial role in timely diagnosis and management [42].

### 1.5 Management

Currently, numerous potential pharmacological therapies are being evaluated in the line of management for VITT. The outcomes range from being propitious to contraindicated or variable in different individuals. Briefed below are specific interventions being employed to overcome VITT.

### 1.6 Intravenous Immunoglobulins (IVIG)

The currently available evidence acknowledges IVIG as a potential treatment depicting remarkable success. Hence it is now incorporated into the treatment regimen. A potential explanation for this involves the Fcγ receptor blockade by the antibodies. The recommended dose in VITT is 1–2 g/kg of the person's body weight. However, ideally, the administered IVIG should be the ones collected before the pandemic. The plausible explanation being vaccine response deterioration due to COVID-19 antibodies present in the donated IGs [43].

### 1.7 Anticoagulants

There has been growing evidence of their efficacy in patients with VITT [44]. In some instances, preliminary trials to validate its effectiveness and progressive clinical worsening in some instances [45] have raised suspicions over its use regarding heparin. Therefore, the American society of hematology (ASH) suggests avoiding the use of heparin unless VITT has been ruled out or another condition diagnosed [42].

The drug of choice is direct oral anticoagulants (dabigatran, apixaban, rivaroxaban, edoxaban, and fondaparinux) or parenteral direct thrombin inhibitors (e.g., bivalirudin and argatroban). The absolute contraindication following anti-coagulation therapy includes a high risk of bleeding. Hence strict clinical monitoring is crucial after initiating oral anticoagulants.

### 1.8 Steroids

Most cases of VITT described steroids as a clinically effective treatment option. However, further data is needed to move past the anecdotal evidence. The above data and prediction are based on their successfully reported usage in our included cases and recently, by Schultz et al. [46], where the combined IVIG and steroids were supported.

### 1.9 Platelet infusion

This therapy is only indicated in significant bleeding. Goel et al. [47] reported a five times increase in mortality of patients infused with platelets following thrombocytopenia. In our included studies, eight reportedly administered platelet infusion. Following Goel et al. [47,48], only two patients survived [49].

### 1.10 Platelet exchange

Plasma exchange was used in three cases. Two out of the three patients survived [50,51]. Relevant details by Garnier et al. were unavailable [14]. Plasma exchange is used in refractory VITT [52]. Clinically, there is insufficient data to evaluate whether plasma exchange can be administered safely in VITT.

Plasma exchange is not a standard treatment option in HIT [42]. Extrapolating this to VITT, we may assume similar effects on patients with VITT. However, more data is required to draw any conclusion.

### 1.11 Aspirin and rituximab

Aspirin or other anti-platelets are currently contra-indicated in VITT due to increased risk of bleeding. Smith et al. [53] suggested a possible prophylactic role of antiplatelets in VITT. This highlights the need for more work in this area.

Rituximab is not recommended currently due to its longer response time (6–8 weeks) [42]. Moreover, this drug's mechanism of action can be explained via its downregulation of CD-20 B-cells. This can potentially lead to the inactivation of antibodies against COVID-19, hence rendering the vaccine administration useless.

### 1.12 Treatment regimen

The following regimen is per the American Society of Hematology (ASH) [42], International Society on Thrombosis and Haemostasis (ISTH) [54], and National Institute for Health and Care Excellence (NICE) in the United Kingdom:

1. Start IVIG.
2. The ISTH guidelines recommend administering steroids if a patient's platelet count is less than 50 × 109/L.
3. Platelet infusion and plasma exchange should not be considered initially.
4. Based on their history and previous clinical profile, patients shall be started an anticoagulant (non-heparin). Vitamin K antagonists should be avoided while the platelet count is low. Moreover, direct thrombin inhibitors should be avoided in pregnant women. DOACs and fondaparinux are suitable for noncritically ill patients.
5. For patients having less than 50 × 103/μL and severe risk of bleeding, IV direct thrombin inhibitors can be used. This will lead to a shorter half-life and rapid action.
6. Fibrinogen levels should be strictly monitored and kept in range (>1.5 g/L)
7. If platelet count remains less than 30 × 109/L despite intravenous immunoglobulin and steroid treatment or fibrinogen level is less than 1 g/L, plasma exchange can be considered after an opinion with hematologists.

## 2 Conclusion

VITT is a rare adverse effect of SARS-CoV-2 vaccination, and the benefits of COVID-19 vaccines continue to outweigh the rare side effects. However, while its incidence is low, there is undoubted an overwhelming need to discern the precise

pathophysiology behind this syndrome to establish proper management protocols. Questions like why certain coronavirus vaccines carry a higher risk than others, why specific individuals develop thrombosis while others don't, higher prevalence in a particular gender and age group, and the impact of different interventions in such patients need to be investigated before a clear conclusion can be drawn. Lastly, future studies must take into consideration both pre-and post-vaccination investigations to discern the role of any underlying condition.

## Ethics statement

Not applicable.

## Funding

None.

## Provenance and peer review

Not commissioned, externally peer-reviewed.

## Please state any sources of funding for your research

None.

## Consent

NA.

## Registration of research studies

Name of the registry: NA
Unique Identifying number or registration ID: NA
Hyperlink to your specific registration (must be publicly accessible and will be checked): NA

## Author contribution

SHA, IU: Study concept or design.

SHA, SW, TGS, NAQ and IU: Data collection, data analysis or interpretation, writing the paper.

ZY: Critical revision of the article.

## Declaration of competing interest

The authors declare that there is no conflict of interests.
